# The Privilege of Suffering

**Published:** 1886-11-06

**Authors:** 


### Words of Consolation.

[Communications for this column should be addressed, "Chaplain," care of the Editor of The Hospital.]

### VI.—The Privilege of Suffering.

At the risk of some repetition upon such an important point, we venture some further remarks this week upon the privilege of suffering as a Christian. We wonder sometimes at protracted and painful sickness, when patience seems to have been tried long enough. The sufferer seems ready, perhaps anxious, to die; yet has to linger and battle on with pain. The same feeling is excited about other trials which are undeserved, so far as we can see. Witness what many good people have to bear on account of the wickedness of others—losses or poverty, violence or aggravation, slander or persecution. Often those who appear to need tribulation least by way of discipline are the very ones chosen for the heaviest burdens. We must go higher, then, than discipline for a solution of the mystery in such cases, and we find an answer in the privilege of suffering. Let us take Job as an early illustration of what God requires of some of His children. Job was as perfect in his way as a character could be for his time. He does not seem to suffer directly for his own sins, though his friends would have it so. Discipline was no doubt necessary for him; but that is not the key to the history. Rather, he was handed over, as it were, to Satan, in order that he might win a great victory for God; and he won it. He established his righteousness by the grace of God, in spite of the very worst the Adversary could do. God seemed to say, in Job's case, to Satan: "Take him, rob him of all that is dear to him; persecute him, plague him with sores; let his friends and neighbours misunderstand and unjustly reproach him; and I will yet prove to you through it all that I can make a creature morally and spiritually superior to the very worst you can do." If this be a fair, and we trust not irreverent, analysis of the Divine intention to be gathered from the Book of Job, the history of that proverbially patient man may be read by sufferers with a view to gleaning from it something more than a lesson upon endurance.

Our blessed Redeemer's sufferings, of course, prove the same point. We recognise the truth clearly enough in His case, because He had no sins of His own to suffer for. He needed no chastisement for the sake of purification; all His sufferings, therefore, belonged to the martyr class, being endured solely for righteousness' sake by One perfectly innocent. For this reason, also, the sacrifice for sin was so complete. But, knowing this well, many of us need to realise that His victory is nevertheless, in one sense, incomplete without ours. We have to fill up the measure of His afflictions for the sake of the Church, if He think well to call us to such an office. St. Paul expressed the purpose of suffering in many a disciple besides himself, when he said: "I now rejoice in my sufferings for you, and fill up that which is behind of the afflictions of Christ in my flesh, for His body's sake, which is the Church" (Col. i, 24).

*(To be continued.)*

